# Incomplete Healing as a Cause of Aging: The Role of Mitochondria and the Cell Danger Response

**DOI:** 10.3390/biology8020027

**Published:** 2019-05-11

**Authors:** Robert K. Naviaux

**Affiliations:** The Mitochondrial and Metabolic Disease Center, Departments of Medicine, Pediatrics, Pathology, University of California, San Diego School of Medicine, San Diego, CA 92103, USA; Naviaux@ucsd.edu; Tel.: +1-619-993-2904

**Keywords:** cell danger response, healing cycle, mitochondria, purinergic signaling, metabokines, sphingolipids, integrated cell stress response, de-emergence, crabtree effect, pasteur effect

## Abstract

The rate of biological aging varies cyclically and episodically in response to changing environmental conditions and the developmentally-controlled biological systems that sense and respond to those changes. Mitochondria and metabolism are fundamental regulators, and the cell is the fundamental unit of aging. However, aging occurs at all anatomical levels. At levels above the cell, aging in different tissues is qualitatively, quantitatively, and chronologically distinct. For example, the heart can age faster and differently than the kidney and vice versa. Two multicellular features of aging that are universal are: (1) a decrease in physiologic reserve capacity, and (2) a decline in the functional communication between cells and organ systems, leading to death. Decreases in reserve capacity and communication impose kinetic limits on the rate of healing after new injuries, resulting in dyssynchronous and incomplete healing. Exercise mitigates against these losses, but recovery times continue to increase with age. Reinjury before complete healing results in the stacking of incomplete cycles of healing. Developmentally delayed and arrested cells accumulate in the three stages of the cell danger response (CDR1, 2, and 3) that make up the healing cycle. Cells stuck in the CDR create physical and metabolic separation—buffer zones of reduced communication—between previously adjoining, synergistic, and metabolically interdependent cells. Mis-repairs and senescent cells accumulate, and repeated iterations of incomplete cycles of healing lead to progressively dysfunctional cellular mosaics in aging tissues. Metabolic cross-talk between mitochondria and the nucleus, and between neighboring and distant cells via signaling molecules called metabokines regulates the completeness of healing. Purinergic signaling and sphingolipids play key roles in this process. When viewed against the backdrop of the molecular features of the healing cycle, the incomplete healing model provides a new framework for understanding the hallmarks of aging and generates a number of testable hypotheses for new treatments.

## 1. Introduction 

Some of the oldest [1], and the most recent [2,3] scientific publications on the biology of aging have focused on nutrition and metabolism as prime drivers. Mitochondria are located at the hub of the wheel of cellular metabolism. The mitochondrial proteome is transcriptionally and post-transcriptionally regulated according to tissue-specific needs [4], consists of about 1300 proteins [5], responds to injury [6], food quality [7], exercise [8], environmental pollution [9], and coordinates the cell danger response (CDR) [10]. The CDR is a term that was coined in 2014 [10], but includes elements of inflammation and healing that have been studied since before the time of Hippocrates (c. 460–370 BCE) [11]. The CDR is an evolutionarily conserved, multi-system response of multicellular organisms that is used to manage and heal from threat or injury. The CDR is a graded response that consists of nested layers that range from the molecular control of electron utilization and cellular oxygen consumption, through changes in the microbiome, mast cells and immune system, to the autonomic nervous system, enteric nervous system, and neuroendocrine circuits that are needed for whole-body integration of the response. When analyzed at the molecular and single-cell level, a widely-studied component of the CDR is known as the integrated cell stress response (ICSR) [12,13,14]. 

## 2. Defining Cellular Stress

In this paper, the word “stress” has a specific scientific meaning. Stress is any force, condition, chemical, pathogen or other stimulus that acts to perturb cellular function, requiring the expenditure of energy and resources to return the cell to its pre-stimulus or to a new steady state. Remarkably, psychological stresses, particularly early life stresses (ELS), regulate some of the same metabolic and gene expression networks used to defend the cell from microbial pathogens, physical injury, poisoning, and adversity of many other types [15,16,17]. In the case of major depressive disorder, innate immune/pro-inflammatory gene expression is increased, while adaptive immune/anti-inflammatory/pro-resolving gene expression is decreased [18]. Greater stresses stimulate proportionately more nucleotide and metabolite release through cell membrane channels [19], leading to greater metabokine and purinergic signaling [20,21]. Learning and development emerge from both conscious stresses and subconscious chemical stresses encountered throughout life. Stresses lead to metabolic memories that help cells and tissues improve future responses to previously encountered conditions [22]. Transient increases in the mitochondrial and cellular sources of reactive oxygen species (ROS) such as superoxide and hydrogen peroxide, reactive nitrogen species (RNS) such as nitric oxide (NO) and peroxynitrite (ONO_2_^−^), reactive aldehydes (RAs), and dissolved oxygen itself, help to regulate cellular redox. Redox, in turn, regulates the efflux of metabolites from the cell via membrane channels and transporters that contain redox-responsive cysteine disulfide residues [23]. Pulses of dissolved oxygen occur with a carbohydrate-rich meal because of the transient inhibitory effect of glucose on mitochondria produced by the Crabtree effect [24]. ROS, insulin, and IL1β are also stimulated naturally by every meal and can vary in magnitude according to the nutrient content of the meal [25]. As the dissolved oxygen concentration rises within the cell, glycolysis becomes inhibited by the Pasteur effect, creating a natural brake on an unchecked inhibition of mitochondrial oxphos from the meal-associated glucose and the Crabtree effect. However, glucose, oxygen, ROS, RNS, and RAs are only a part of the multi-faceted metabolic signaling network that is used to control cellular reactivity, epigenetic marking, and gene expression. Over 100 chemosensory G-protein coupled receptors respond to metabokines and peptides released after stress and injury and regulate the healing cycle [11].

## 3. The Healing Cycle

**Figure 1:**
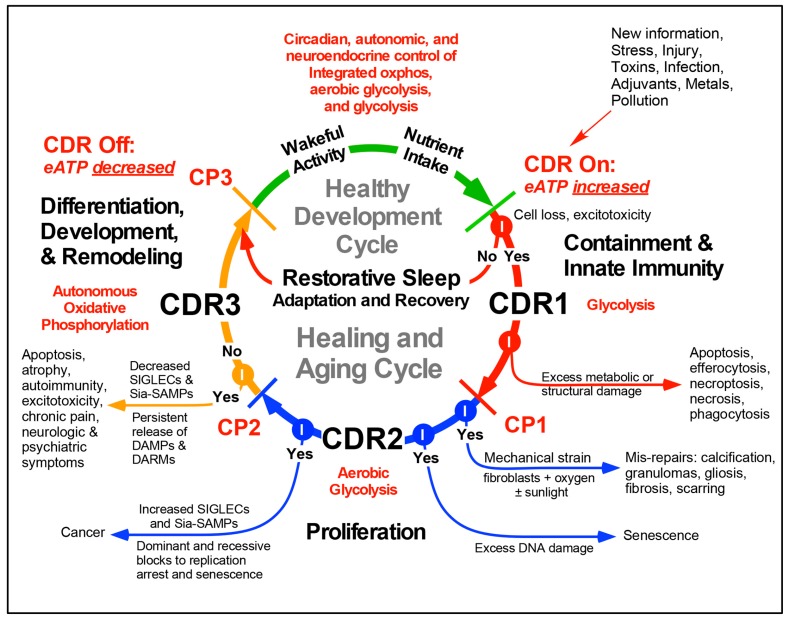
The metabolic features of the health and healing cycles. Abbreviations: CDR—cell danger response, eATP—extracellular ATP, CP1-3—checkpoints 1, 2, and 3, DAMP—damage-associated molecular pattern, DARM—damage-associated reactive metabolites, SIGLEC—sialic acid binding immunoglobulin-type lectin, e.g., CD33-related SIGLECs (CD33r-SIGLECs), Sia-SAMP—sialoglycan self-associated molecular pattern.

The mitochondrial responses that initiate and maintain the CDR are used to control cellular bioenergetics, oxygen utilization, redox signaling, and metabolism needed for healing [6] and regeneration [26] after injury or threat. The stages of the CDR are illustrated in Figure 1. Wakeful activity with nutrient intake, followed by restorative sleep are essential parts of health. These activities stimulate an integrated mix of three metabolic states that are controlled locally by metabolic signaling, and systemically by the central nervous system (CNS). Healthy whole-body function requires the coordination and use of cell-specific (1) glycolysis, (2) aerobic glycolysis, and (3) oxidative phosphorylation for energy and metabolism (Figure 1). Chemical activity associated with nutrient intake, brain activity, and basal metabolism, and added physical activity from natural child play or adult exercise lead to the graded release of extracellular ATP and related nucleotides, and to glutamate release [27,28] through stress-gated P2X7-pannexin and other channels in the cell membrane [19]. Once outside the cell, ATP and related nucleotides participate in purinergic signaling. Receptor binding to ATP and ADP leads to IP3-gated intracellular calcium release [29] and contextual changes in gene expression through autocrine and paracrine signaling pathways [30]. After extracellular metabolism by CD73 and CD39, ATP is converted to ADP, AMP, and to adenosine that acts to inhibit excess chemical stimulation and plays a key role in initiating and maintaining sleep by binding to P1/Adenosine /ADORA A2AR and A1R receptors [31]. This is illustrated as the restorative sleep cycle in Figure 1.

**Figure 2:**
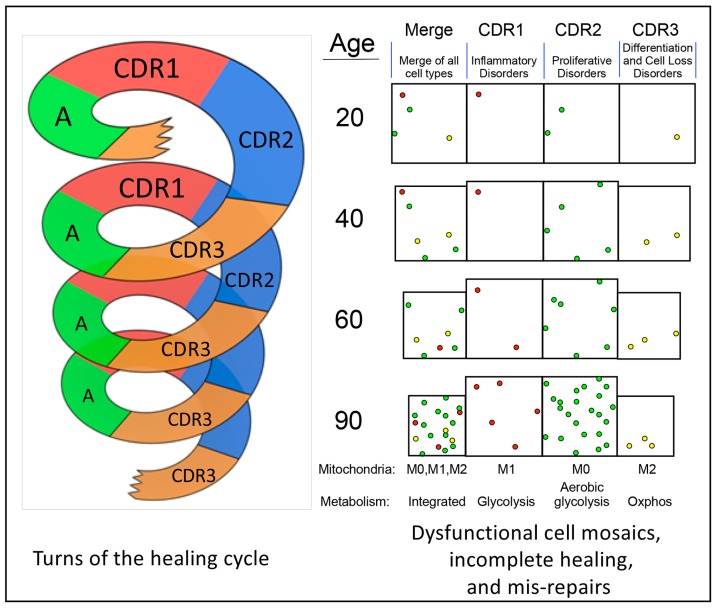
Repeated cycles of incomplete healing lead to aging and age-related disease. The spiral represents sequential turns of the healing cycle throughout life. Colored cells in the boxes on the right represent cells that have been delayed or arrested in a stage of the healing cycle. The decreased size of some boxes represents the loss in tissue volume from cell loss and atrophy. In this example, most arrested or delayed cells in the merge on the right after 60 and 90 years are in CDR2 (green). This will create an increased risk of proliferative disorders such as diabetes, heart disease, and cancer. Color code: CDR1 cells—red; CDR2 cells—green; CDR3 cells—yellow. Abbreviations: A—wakeful activity and nutrient intake. M1—mitochondria adapted for cell defense, reactive oxygen, nitrogen, and aldehyde production; M0—mitochondria adapted for cell growth and Warburg metabolism; M2—mitochondria adapted for oxidative phosphorylation (oxphos).

**Figure 3:**
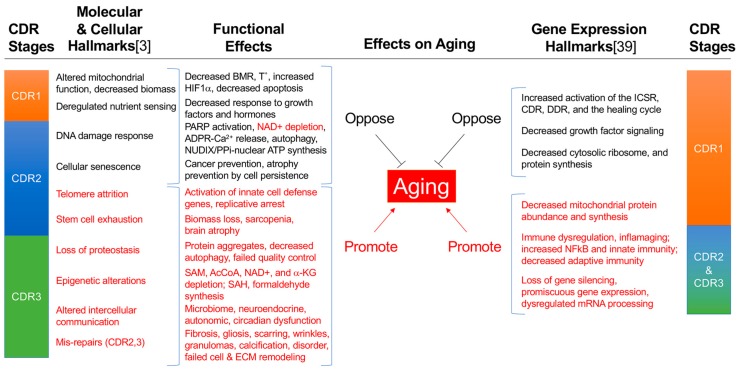
The hallmarks of aging as natural products of incomplete healing and the cell danger response [3,39]. Black font: hallmarks that oppose aging. Red font: hallmarks that promote aging. Abbreviations: CDR—cell danger response, ICSR—integrated cell stress response, DDR—DNA damage response, BMR—basal metabolic rate, T°—basal body temperature, HIF1α—hypoxia inducible factor 1α, PARP—poly ADP ribose polymerase, NAD+—nicotinamide adenine dinucleotide, ADPR—adenosine diphosphate ribose, SAM—S-adenosyl methionine, AcCoA—acetyl CoA, α-KG—alpha ketoglutarate, SAH—S-adenosyl homocysteine, PPi—pyrophosphate, NUDIX—nucleoside diphosphate X hydrolases, e.g., NUDT5, ECM—extracellular matrix.

When the stress is of sufficient magnitude to cause cell death, more ATP is released and acts as a pro-inflammatory damage-associated molecular pattern (DAMP). Increased extracellular ATP triggers entry into the CDR1 stage of the healing cycle (Figure 1). Once the CDR is triggered, three different metabolic stages must be activated in sequence to heal. Healing cannot occur without activation of this metabolically-controlled cycle. CDR1 is characterized by the upregulation of anaerobic glycolysis and a reallocation of cellular resources for defense, damage containment, innate immunity, and repair at the expense of normal differentiated tissue function. Gap junctions between cells are decreased or lost as the tissue structure is disrupted by injury, and cell-autonomous functions become primary and metabolic cooperation between neighboring cells is decreased or suspended. Platelets and neutrophils are recruited to sites of injury or infection. CDR2 uses aerobic glycolysis to support stem cell recruitment and cell division needed for biomass replacement of cells lost in CDR1. If cell loss is not replaced, then age-related atrophy and sarcopenia occur. If excessive DNA damage is sustained by a cell in CDR2, replicative senescence occurs [32]. If the blocks to senescence are broken, then cancer can occur. If oxygen levels are high and significant mechanical strain is present, tissue fibrosis or wound scarring is stimulated (Figure 1). Fibrosis is one of several different types of mis-repairs that contribute to the symptoms of aging [33,34,35] (Figure 1, Figure 2 and Figure 3). 

In CDR3, cell-autonomous, aerobic metabolism by mitochondrial oxidative phosphorylation is gradually restored as new cells born in CDR2 take up residence in the recovering tissue and establish new tissue-specific contacts and gap junctions needed to extinguish the gene expression programs used for defense and growth in CDR1 and CDR2, and to restore normal differentiated cell, tissue, and organ function. If damage-associated molecular pattern (DAMP) and damage-associated reactive metabolite (DARM) release persists [36] or if ROS production is extinguished prematurely [37], autoimmunity can develop [38]. The adaptive immune response is regulated in CDR3. Persistent DAMP and DARM release can stimulate excitotoxicity in cells still in the CDR1 and CDR2 stages within a dyssynchronous mosaic of healing cells. With the completion of CDR3, the concentration of metabokines and DAMPS in the extracellular space is actively reduced to levels compatible with restoration of cell specialization and reintegration of cells back into a metabolically optimized cellular network. Re-integration and re-specialization are necessary for cells that have recently exited the CDR in order to re-establish their responsiveness to circadian patterns of wakeful activity and restorative sleep (Figure 1). 

The sequence and stages of the healing cycle are highly conserved and tightly choreographed. Once pathological stress or cell death occurs, the same stages of the healing cycle are activated and restore normal function after any recoverable injury (Figure 2). Inevitably, some cells fail to complete the healing cycle and are left behind with each turn of the cycle. These arrested or delayed cells are unable to return to a normal metabolism and the gene expression pattern that is needed for peak differentiated cell and organ function (Figure 2). As non-specialized cells and mis-repairs such as fibrosis accumulate in incomplete stages of the healing cycle, the peak performance of tissues is degraded over time, and the risk for age-related disease is increased. 

## 4. Three Functionally-Polarized Forms of Mitochondria Are Used by the CDR

For over 60 years, mitochondria have been thought of as “damaged” or “dysfunctional” if they shift from energy production by oxidative phosphorylation to ROS production or shift from burning carbon skeletons to CO_2_ and water to synthesizing new carbon skeletons for export as building blocks needed for cell growth. Yet mitochondria shift regularly and necessarily between these states throughout life. A simplified way of understanding the alternative differentiation states of mitochondria is to see the organelles as the metabolic gate-keepers of the electrons harvested by breaking down the carbon–carbon bonds in food. In this scheme, electrons can be used in mitochondria: (1) to fully reduce oxygen (O_2_) to water (H_2_O) while capturing the released chemical energy to make ATP by oxidative phosphorylation in the third stage of the cell danger response, CDR3 and in health, (2) to partially reduce oxygen to superoxide radicals (O_2_^−^) and hydrogen peroxide (H_2_O_2_) while relying on anaerobic glycolysis for ATP synthesis in CDR1 and cell defense, or (3) to use the electrons to synthesize new carbon–carbon bonds to produce and export building blocks such as citrate for cell membrane lipid synthesis or orotic acid for pyrimidine synthesis, while still consuming oxygen to make water and making ATP by aerobic glycolysis in CDR2 and cell growth (Figure 1). 

A new nomenclature was introduced in 2017 to describe these three, functionally-polarized forms of mitochondria of the CDR [11,29]. All three “species” of mitochondria co-exist in different proportions in cells throughout development and aging in all multicellular organisms. The transition between states is determined by the interaction between nutrition and metabolism, the developmental and chronological age of the organism, the recent state of cell danger signaling and healing, and ambient environmental conditions. The naming of the differentiation states of mitochondria was based on the recognition that the pro-inflammatory state of macrophages known as M1 macrophages corresponded with the ROS producing capacity of their mitochondria and energy production by glycolysis [40]. The anti-inflammatory/pro-resolving M2 macrophages were found to contain mitochondria adapted for oxidative phosphorylation (oxphos). M0 macrophages are not yet committed to a fully M1 or M2 phenotype and contain mitochondria that are intermediate between M1 and M2. In the healing cycle, three different mitochondrial differentiation states are used to meet the specialized needs of each stage of the CDR. M1 mitochondria are used in CDR1. M0 mitochondria are used in CDR2, and M2 mitochondria are used in CDR3 (Figure 1 and Figure 2, Table 1).

In vitro protocols have recently been developed that distinguish between M1, M0, and M2 macrophages experimentally based on mitochondrial phenotypes [41]. M1 mitochondria consume small amounts of oxygen, have a low spare respiratory capacity (SRC, or physiologic reserve capacity), can use fatty acids for ROS production and NLRP3 assembly [42], are not dependent on glutamine, and the cells containing M1 mitochondria produce large amounts of lactic acid. In contrast, M2 mitochondria consume more oxygen at baseline, can use both glucose oxidized to pyruvate and fatty acids for oxphos, have a higher SRC, are dependent on glutamine for the fully differentiated phenotype, and the cells produce small amounts of acid. M0, uncommitted or multipotential mitochondria have a low basal oxygen consumption similar to M1, shunt some glucose down the pentose phosphate pathway (PPP) for NADPH and building block production, have an SRC that is intermediate between M2 and M1 organelles, and produce small amounts of extracellular acid, similar to M2 [41] (Table 1). 

M0, M1, and M2 mitochondrial phenotypes also occur in solid tissues and can be recognized in part by morphological criteria. Mitochondria can be described informally as being distributed along a spaghetti (filamentous) and meatball (punctate) gradient. M2 mitochondria are filamentous and interconnected, and predominate in post-mitotic or slowly regenerating tissues. M1 mitochondria are punctate, and M0 mitochondria are intermediate in form, with both short filaments and punctate organelles reminiscent of the coccobacillary forms of their proteobacterial ancestors [44]. Mitochondrial fusion–fission dynamics naturally regulate the balance between fused and elongated mitochondria, and fragmented/fissioned mitochondria according the growth and metabolic characteristics of the cell [45]. M0, uncommitted, or stem-like mitochondria predominate in cells that survive after exposure to chemotherapeutic agents or toxins [46]. M2 mitochondria under these conditions are depleted by DRP1-dependent fission/fragmentation, and conversion to M1 organelles prior to cell removal by apoptosis. Similar transitions in mitochondrial structure and function are readily demonstrated in brain astroglia before and after treatment with pro-inflammatory triggers such as lipopolysaccharide (LPS) and interferon gamma (IFN-γ) [43].

The total mitochondrial biomass is decreased naturally after triggering the cell danger response (CDR) with toxins [46], physical injury [6], infection, or any trigger that activates the healing cycle [11]. Mitochondrial biomass can also be reduced experimentally by the dominant-negative expression of the D1135A allele of the mitochondrial polymerase gamma (POLG1) to deplete mitochondrial DNA copy numbers by about 50% [47]. This mouse model mimics the mitochondrial defects that are a well-established hallmark of aging [48]. The phenotypes of aging found in this model included increased NFkB and matrix metalloproteinase 9 (MMP9) expression, increased inflammation, age spots, wrinkled skin, and premature hair loss. These transcriptional and anatomical signs of aging were reversed within 1 month of treatment in mice by unblocking mtDNA synthesis and restoring normal mtDNA copy numbers [47].

## 5. The Importance of Nucleotides and Purinergic Signaling

Of an estimated 1300 mitochondrial protein coding genes, 1158 are catalogued in MitoCarta v2.0 [5]. Only 13 mitochondrial proteins are encoded by mitochondrial DNA (mtDNA). The remaining 1145 are encoded by nuclear genes that are subject to tissue-specific gene expression programs. At least 789 mitochondrial proteins (68% of 1158) are enzymes with catalytic functions that have been assigned enzyme commission (EC) numbers, or encode subunits of multi-protein enzyme complexes such as those in respiratory chain complexes I, II, III, IV, and V, or are transporters, or kinases that use ATP (Table S1). At least 433 (55% of 789) of these proteins are regulated by the availability of purine and pyrimidine nucleotides such as ATP, GTP, UTP, NAD(P)+, or FAD either as substrates, or as allosteric regulators (Table S1). No other single class of molecules regulates more of the mitochondrial proteome than the nucleotides. In addition, an unknown fraction of nuclear mitochondrial genes is regulated transcriptionally and post-transcriptionally by purinergic signaling via the 12 G-protein coupled receptors (GPCRs; 4 P1R and 8 P2YR) and seven ionotropic receptors (P2XR) that are widely distributed in all tissues [49]. Some purinergic receptors are also expressed in intracellular compartments such as mitochondria, lysosomes, and the nucleus [50]. For example, P2X6 receptors are translocated to the nucleus in an age-dependent manner, interact with mRNA splicing factor 3A1, decrease mRNA processing, and contribute to aging [51]. 

## 6. The Importance of Nutrition in Healing and Aging

Different stages in child development, human aging, and athletic performance have different specific nutrients and calories that produce the best outcomes at each stage [52,53,54]. Based on measured differences in mitochondrial substrate preferences between M1, M0, and M2 organelles (Table 1), it is hypothesized that each stage of the healing cycle—CDR 1, 2, and 3 in Figure 1, and even certain chronic illnesses stuck in one of the three stages of the CDR [11]—will also have different stage-specific nutritional needs for optimal outcomes. Both chemical mass action from substrate supply and metabolite signaling via metabokines [11] are likely to be important mechanistically. Some of the broadest clues that connect nutrition with aging, mitochondria, and healing come from studies of caloric restriction [3]. Natural aging results in a reduction in the rate of turnover of mitochondria (mitochondrial biogenesis, or “mitochondriogenesis”), and a decrease in mitochondrial protein synthesis, standing mitochondrial biomass, respiratory reserve capacity, and oxphos function [55] (Figure 3). Caloric restriction, on the other hand, stimulates mitochondrial turnover through pathways associated with AMPK-stimulated autophagy and mitophagy [56]. This leads to improved mitochondrial quality control, oxphos function, and reserve capacity, but does not increase overall mitochondrial biomass measured as mtDNA copy number [57]. Caloric restriction also leads to a decrease in circulating thyroid hormone, decreased resting energy expenditure (REE), and a decrease in circulating anabolic hormones such as IGFI, insulin, human growth hormone, and testosterone [58]. From an evolutionary point of view, this hypometabolic response allows fewer calories to be consumed while opposing weight loss during periods of seasonal hardship. 

Caloric restriction is a classical trigger of a reversible, stress-resistant, non-reproductive stage in the nematode *Caenorhabditis elegans* called dauer. Dauer has been the source of discovery of many longevity genes and has stimulated a productive discussion of the difference between lifespan and healthspan extension [59]. Dauer permits animals to live for up to 4 months under harsh conditions instead of their normal lifespan of 2 weeks. However, longevity in dauer comes at the cost of much reduced function and many changes associated with altered sensory anatomy [60], repetitive behaviors [61] and metabolism [62]. When calories are restored to animals in dauer, they exit the stage and re-enter their normal life-cycle, picking up where they left off, as if little or no biological aging had occurred during the time they spent in dauer [63]. 

More specific changes in nutrient supply also play an important role in aging and healing. Dietary supplementation with branch chain amino acids at a level of 1.5 g/kg/day, corresponding to about 1% of daily calorie intake in mice, has been shown to extend lifespan by about 10% [64]. This effect required normal production of nitric oxide (NO) by endothelial nitric oxide synthase (eNOS) [64]. The selective addition of a stoichiometric mix of essential amino acids (EAAs) including branch chain amino acids, also has anti-cancer effects by promoting growth inhibition and apoptosis in transformed but not in normal cells [65]. EAAs also promoted wound healing by moderating inflammation in the early stages, and maintaining TGFβ needed for tissue remodeling in the later stages of healing [66]. This later effect is in what would be called CDR3 in the model presented here (Figure 1). Interestingly, TGF β (DAF-7) signaling also plays a key role in recovery from dauer and the re-establishment of normal development in *C. elegans* [67].

## 7. Progressively Dysfunctional Cellular Mosaics

The number of nucleated cells in a 70-kg adult male is about 5 × 10^12^. Non-nucleated red blood cells are 5-times more abundant (2.5 × 10^13^), but make up just 6.5% of the total mass of an adult [68]. The total number of human cells in an adult is 3.0 × 10^13^. There are also 3.8 × 10^13^ bacterial cells in a typical adult body [68]. Cells die every day and must be replaced. It is estimated that an adult turns over about 1 × 10^10^ (10 billion; about 10 grams of) cells/day by apoptosis [69]. This is equivalent to about 1 in 500 nucleated cells/day removed by apoptosis. The distribution of cell turnover is highly heterogeneous. Most spontaneous, or physiologic cell death with replacement occurs in short-lived cells such as those in bone marrow, intestines, skin, and hair follicles whose function is tolerant to spatial changes in tissue architecture produced by continuous cell growth and use-dependent removal or exit of cells from the compartment of origin. These rapidly dividing and structurally malleable tissues can turn over several times per year, with nearly 100% of the cells in these compartments turning over in a few days to weeks or months. Other tissues turn over more slowly. Adipose tissue is replaced at a rate of 10% of the cells per year [70], turning over completely in about 10 years. Skeletal muscle turns over at a rate of 6.6% per year [71]. Muscle fiber loss accelerates after age 60, leading to sarcopenia [72]. The kidneys weigh about 150 grams each, shed about 1.7 × 10^6^ epithelial cells/day into the urine [73], and replace this loss and other losses by cell division and remodeling from recruited stem cells [74]. The adult liver weighs about 1400 grams and consists of about 2.4 × 10^11^ cells and replaces about 1.8% per year as a young adult, but fewer as liver size decreases with age [75]. Heart cells are replaced at a rate of 1% per year at age 25, falling to 0.45% per year at age 75 [76], and pancreatic islet β-cells are long-lived and not replaced after age 30 years [77]. 

Exposure to physical injury, toxins, or infection adds pathological cell death to the basal level of physiologic cell death described above. When a cell dies physiologically by apoptosis, the inflammatory reactions associated with CDR1 are avoided. Instead, the tissue skips to CDR2 and CDR3 to replace the lost cell and restore normal metabolism and tissue-specific gene expression patterns (Figure 1). The number of cells that die pathologically during a typical viral or bacterial infection will be dependent on the type of microbial pathogen and the severity of the infection, but the exact cell numbers lost in the course of a typical infection are not known. An average child has 5–6 recognized viral or bacterial infections each year for the first 5 years of life, then 2–3 per year throughout adult life [78]. Pathological cell death caused by infection, toxins, or injury will trigger inflammation and entry into the healing cycle by activating CDR1. As a thought experiment, one can imagine a systemic viral infection that might kill 1 × 10^10^ cells (about 10 grams, or 2 teaspoons), a number equal to the basal loss per day by apoptosis. Somatic DNA mutation rates are about 2.7 × 10^−5^ per typical 10,000 bp, protein-coding locus per cell division [79]. In this example, one turn of the healing cycle would result in 270,000 cells (10^10^ cells replaced × 2.7 × 10^−5^ mutations/gene/division = 270,000 cells) sustaining a mutation that marks that cell as different from neighboring cells in the tissue. Mitotic recombination errors and chromosomal microaneuploidy [80], mobilization of retroelements [81] and endogenous retroviruses [82] in recruited stem cells, and reactivation and suppression cycles of latent DNA virus infections [83] will contribute to genetic variation produced by repetitive activation of the CDR over a lifetime. In addition to DNA mutations, there will also be an even larger number of induced and stochastic changes in transcription, post-transcriptional, and metabolic features that lead to changes in cell development. 

With each turn of the healing cycle, cells such as neutrophils move out of capillaries and into tissues, releasing ROS via activated NADPH oxidases such as NOX2. Over time, this cyclic process creates tides of reactive oxygen, metabolic, innate immune, and inflammatory defenses that rise with injury and fall during wellness, ebbing and flowing with each turn of the healing cycle. Sialic acid binding immunoglobulin-type lectins (SIGLECs) include the CD33-related molecules expressed on neutrophils, natural killer (NK), macrophages, and T-cells that modulate ROS production, innate immune cell, and adaptive T-cell activity upon binding to sia-SAMPs (sialoglycan self-associated molecular patterns) [84]. Across species, the number of CD33r-SIGLEC genes is associated with healthy aging in the “wellderly”, and decreased numbers accelerate age-related symptoms in mouse models [85]. Many cancers emerge from hypersialated cellular fields that have the effect of dampening the immune response by engaging inhibitory SIGLECs such as Siglec-9 [86]. The ceaseless tides or waves of innate immune cell migration into and out of tissue compartments continues throughout life with each turn of the healing cycle (Figure 1 and Figure 2), and contributes to the progressively dysfunctional mosaics illustrated in Figure 2. 

In the brain, a compensatory system of glial–lymphatic (glymphatic) tidal flows occurs by regulated changes in cell volume that are controlled by circadian changes in metabolism [87]. This daily cycle of glympahtic flow helps to remove aggregates of tau and beta amyloid that would otherwise accumulate as the byproducts of CDR activation both from normal learning and from microglial and synaptosomal innate immune activation that increase progressively with aging. Interestingly, a number of molecules that regulate mTOR and other metabolic aspects of the CDR, have recently been found to slow the aging process and extend longevity, while simultaneously protecting against age-related markers of Alzheimer dementia in animal models [88]. These “geroneuroprotecting” drugs were plant-based natural products similar to curcumin and polyphenols such as fisetin. The healing cycle-promoting properties of these drugs prevented the accumulation of tau and beta amyloid markers of Alzheimer dementia even though the drugs were not directed at the protein markers specifically [88]. 

The importance of innate immune activation and healing in aging is underscored by a recent discovery in Werner syndrome. Werner syndrome is a recessively inherited adult progeria syndrome caused by mutations in the Werner helicase (WRN). In addition to genomic instability caused by WRN mutations, the protein was recently found to play a key role in innate immunity as a transcriptional coactivator of the NFkB-dependent expression of the chemokine IL8 [89]. Werner mutations inhibit NFkB- and IL8-dependent ROS production. ROS production is not only an essential component of an effective CDR1 (Figure 1), but is important for NRF2 induction of long-term anti-oxidant and detoxification defenses [90]. In addition, the WRN protein is important for HIF1 stabilization [91] needed for tissue regeneration [26] during CDR2 (Figure 1). 

With each turn of the healing cycle, the induced combination of transcriptional and metabolic changes and the DNA damage response will result in some injured and newly replaced cells being unable to complete the normal stages of the cycle. Perhaps 0.1–1 million cells of the 10^10^ cells that must be replaced after pathological cell death may be left behind, stuck or delayed in one of the three stages, CDR1, CDR2, and CDR3. An even larger number of cells whose function was transiently changed by injury, but were not killed, will contribute to the cells that are left behind in stages of the healing cycle. This process is illustrated as progressively dysfunctional mosaics on the right of the spiral in Figure 2. The ecogenetic interaction of inherited genotype of a given individual with the particular environmental insult (nutritional stress, pathogen, toxin, or physical injury) will determine how many cells are delayed or lost in the three different stages of the CDR. Examples of aging cellular mosaics are illustrated at age 20, 40, 60, and 90 years (Figure 2). Delayed and arrested cells that are incompletely differentiated create physical and metabolic separation—buffer zones—that act like control rods in a nuclear reactor that absorb signals and inhibit communication between previously adjoining, synergistic, and metabolically interdependent cells. Loss of cellular connectivity through functional gap junctions decreases the physiologic reserve capacity and increases vulnerability to stress-related cell death [92].

The propagation of calcium waves from cell to cell in a tissue via the inositol trisphosphate (IP3)-gated release of intracellular calcium stores is a common example of the cooperative response to neuroendocrine signaling, and is highly dependent on fully differentiated mitochondrial function [93]. CD38 increases with age [94] and is used by the cell to synthesize cyclic adenosine diphosphate ribose (cADPR) from NAD+ and nicotinic acid adenine dinucleotide phosphate (NAADP) from NADP+ as IP3-responsive calcium signaling declines [95]. cADPR and NAADP are intracellular ligands that stimulate IP3-independent release of calcium from the endoplasmic reticulum (ER) and lysosomes, respectively [29]. NAD+ and NADP+ are depleted by CD38 in the course of aging [94]. Cellular depletion of NAD+ and NADH leads to a decrease in mitonuclear communication [96], and to progressive declines in mitochondrial electron transport that contribute to aging [97]. Supplementation with the NAD+ precursor, nicotinamide riboside (NR), in animal models improves cell differentiation, restores laminin scaffolding and a more normal cellular mosaic in tissues, attenuates senescent cell formation, and increases longevity by about 10% [98]. In a recent study of the effect of transplanted senescent cells, the authors found that ≥ 1 senescent cell among 10,000 total cells in the body (0.01%), or ≥ 1 in 350 normal cells in a specific tissue (≥0.28%) was enough to produce a dysfunctional cellular mosaic and measurable functional defects such as decreased grip strength and exercise capacity [99].

## 8. De-Emergence as a Cause of Dysfunction

Complex living systems are comprised of at least seven discrete subsystems: (1) molecules; monomers, metabolites, and other building blocks, (2) polymers requiring energy for synthesis, such as proteins, polysaccharides, nucleic acids, and lipids made of amino acid, monosaccharide, nucleotide, acetyl and isoprene monomers, respectively, (3) polymers that assemble spontaneously such as charged and neutral lipids and hydrophobic proteins that form membranes, lipid droplets, and other lowered free energy structures in aqueous matrices, (4) organelles, (5) cells, (6) tissues, (7) organs. Many of the phenotypes associated with normal development, health, disease, and aging are emergent properties that depend on the function, arrangement, and interaction of the subsystems, but are qualitatively, quantitatively, and chronologically distinct from any single subsystem. For example, long-term memory is an emergent property that requires new protein synthesis that alters several aspects of all the subsystems that make up the brain, but knowledge of any one of the subsystems is insufficient to explain long-term memory. Other emergent properties of health include the ability to walk, talk, think, eat, excrete, mount an immune response, reproduce, detect and respond to a toxin or injury, and to heal. Aging degrades each of these abilities. If health is thought of as a collection of emergent phenotypes, then illness and aging can be thought of the gradual fading away, or de-emergence of the emergent properties that results from the degradation of metabolic and cellular order and the interactions of the subsystems that define health and resilience to environmental change. The images of progressively dysfunctional cellular mosaics shown in Figure 2 illustrate just one level—the cellular level—of the problem. Dysfunctional mosaics occur in every one of the seven subsystems, and mis-repairs accumulate at each level with each turn of the healing cycle. 

## 9. The Hallmarks of Aging Emerge as a Result of Incomplete Healing

The molecular, cellular [2,3,48] and gene expression hallmarks [39] of aging can be organized according to their usage during the healing cycle and the stages of the multisystem CDR [10,11,29] (Figure 3). These hallmarks have been identified in studies of aging that have led naturally to an improved molecular understanding of mitochondrial stress [13,17,100], the integrated cell stress response (ICSR) [12,101,102], and the CDR [10,11,29,103]. Each trait associated with these hallmarks arises naturally from the activation of a molecular feature needed in the healing cycle, followed by the failure to extinguish this normal function or gene expression state of the CDR once it is no longer needed. Persistence of an aging hallmark after a turn of the healing cycle (Figure 2) can also occur in five other ways: (1) when a cell sustains too much genetic damage and becomes senescent, (2) when a cell that has been removed is not replaced and leads to tissue atrophy, (3) when a cell is replaced but fails to re-specialize according to the differentiated needs of the tissue, (4) when a mis-repair such as fibrosis or scarring [104] cannot be removed by remodeling, and (5) when genetic and metabolic changes bypass senescence and lead to cancer (Figure 1). The legacy of each of these events is to add to the dysfunctional mosaic illustrated in Figure 2. 

Brain, muscle, nerve, and endocrine tissues are particularly susceptible to the incomplete replacement of lost cells once they have died and have been removed because of their limited regeneration capacity and the high degree of spatial organization required for optimal organ function. The function of these tissues is highly dependent on the spatial organization and metabolic complementarity of the cellular mosaic. This complementarity can only be achieved through cell specialization and chemical cooperativity between neighboring cells, which in turn, depends on the relative position and architecture of layers and columns of cells that must remain fixed over long periods of time. The cost of removing a cell from a chain of connected cells in the cerebral cortex or any other part of the brain has a high price that cannot easily be repaid by recruiting stem cells from another location. However, the cost of converting a cell to the hypersecretory phenotype of a senescent cell [105] is even higher, since just one senescent cell in 350 normal cells is enough to decrease the function of a tissue [99]. The smaller collective volume of cells that occurs in most tissues with age is illustrated in Figure 2. The decreasing volume of tissues in the columns labeled “Merge” and “CDR3” represents the effect of cell loss (atrophy) that occurs with incomplete progress through the healing cycle (Figure 1 and Figure 2). In the example illustrated in Figure 2, more cells have accumulated in CDR2 (illustrated in green) by the ages of 60 and 90 years. Accumulation of cells in CDR2 will increase the risk of diseases such as diabetes, heart disease, and cancer because cells arrested or delayed in CDR2 maintain their proliferative capacity [11] (Figure 1). Proliferative capacity is maintained in part because cells in CDR2 contain more multipotential M0 mitochondria adapted for Warburg metabolism (Table 1). Inherited mutations in the RECQL4 helicase, which interacts with p53 and POLG in mitochondria, increase aerobic glycolysis associated with CDR2, lead to an increased risk of cancer, and to a form of progeria known as Rothmund–Thomson syndrome [106,107,108]. 

## 10. Conclusions

A new model is presented that reframes aging as the result of repeated cycles of incomplete healing. In this model, cycles of incomplete healing stack over time, leading to cellular mosaics that become progressively dysfunctional with age (Figure 2). Cells that accumulate in one of the three stages of the cell danger response (CDR) lead to specific risks of age-related disease. The accumulation of cells in CDR1 leads to chronic inflammatory and pain syndromes, and to susceptibility to chronic viral, bacterial, and fungal infections that require adaptive T-cell immunity for eradication [11]. The accumulation of cells in CDR2 leads to diabetes, heart disease, congestive heart failure, peripheral vascular disease, fibrotic disorders, and cancer risk. The accumulation of cells in CDR3 leads to autoimmune disorders, immune suppression or deficiency, neuropathic pain syndromes, behavioral and mental health disorders, or neurodevelopmental and neurodegenerative disease [11]. It is currently unknown what drugs, devices, or procedures can unblock arrested cells in the mosaic and permit the completion of the healing cycle. However, specific nutritional interventions such as nicotinamide riboside or essential amino acids have been found to facilitate healing [66], longevity [98] or both [64,66] in animal models. A prediction of the incomplete healing model is that differentiation checkpoints exist at each stage of the CDR (Figure 1) and that the molecular signals that are used naturally by cells to facilitate completion of healing may provide fresh clues for both preventing and treating age-related symptoms and disease. One procedure that is well-known to improve physical and psychological well-being, decrease mortality, and decrease the risk of age-related disease, is exercise [109,110]. The effects of exercise are multifaceted, but the increase in autophagy and remodeling of the mitochondrial network, leading to adaptive improvements in quality control and increased reserve capacity, may play key roles [111]. The search for geroneuroprotectants [88] and chemical exercise mimetics [112] has already begun. However, a deeper understanding of the role of natural metabolites as signaling molecules—metabokines [11]—and molecules regulated by exercise—exerkines and exosomes [113,114,115]—that regulate the completion of the healing cycle holds promise for preventing, slowing, and perhaps reversing [47] some of the effects of aging. Interventions that exploit purinergic [20] and sphingolipid [116] signaling pathways may be particularly powerful since over half of all mitochondrial proteins are regulated by ATP, NAD+, and related nucleotides (Table S1), and many of the molecular hallmarks of aging trace to or regulate the interplay between purines, mitochondria, sphingolipids [117,118], and the nucleus [119]. 

## Figures and Tables

**Table 1:** Phenotypic characteristics of animal cell mitochondria.

No.	Trait	Mitochondrial Phenotype [40,41,43]		
		M0	M1	M2
1	Cellular energy metabolism	Aerobic glycolysis	Glycolysis	Oxidative phosphorylation
2	Mitochondrial DNA copy number	Intermediate	Low	High
3	Predominant morphology	Intermediate	Punctate	Filamentous
4	Cell replicative potential	High (Warburg)	Intermediate	Low
5	Cell multilineage regenerative potential	High	Low	Low
6	Cell differentiation potential	Low	Intermediate	High
7	Cell cancer potential	High	Intermediate	Low
8	Inflammatory potential	Intermediate	High	Low
9	Cell susceptibility to killing by apoptosis	Low	Intermediate	High
10	Inducible organellar quality control	Intermediate	Low	High
11	Baseline oxygen consumption	Low	Low	High
12	Stressed (uncoupled) oxygen consumption above baseline (spare respiratory capacity)	Intermediate	Low	High
13	ROS production	Intermediate	High	Low
14	NLRP3 inflammasome assembly	Low	High	Low
15	Lactate release from cells	Intermediate	High	Low
16	Pentose phosphate pathway (PPP)	High—NADPH for biosynthesis and cell growth	Intermediate—NADPH for NOX	Intermediate—NADPH for redox
17	Use of fatty acid oxidation (FAO)	Fatty acid synthesis for growth > FAO	For ROS and NLRP3 activation	For oxphos
18	Use of glucose	Glycolysis and PPP	Glycolysis and lactate release	PPP and pyruvate for oxphos
19	Use of glutamine	High: citrate for ATP citrate lyase and Acetyl-CoA	Low	High: oxphos via alpha-ketoglutarate
20	Stage of greatest use in the healing cycle and cell danger response	CDR2	CDR1	CDR3

## Supplementary Materials

The following are available online at https://www.mdpi.com/2079-7737/8/2/27/s1, Table S1: The mitochondrial proteome and nucleotide regulation.

## References

[1] McCay C.M., Crowell M.F. (1934). Prolonging the life span. Sci. Mon..

[2] Finkel T. (2015). The metabolic regulation of aging. Nat. Med..

[3] Lopez-Otin C., Galluzzi L., Freije J.M.P., Madeo F., Kroemer G. (2016). Metabolic Control of Longevity. Cell.

[4] Pagliarini D.J., Calvo S.E., Chang B., Sheth S.A., Vafai S.B., Ong S.E., Walford G.A., Sugiana C., Boneh A., Chen W.K. (2008). A mitochondrial protein compendium elucidates complex I disease biology. Cell.

[5] Calvo S.E., Clauser K.R., Mootha V.K. (2016). MitoCarta2.0: An updated inventory of mammalian mitochondrial proteins. Nucleic Acids Res..

[6] Naviaux R.K., Le T.P., Bedelbaeva K., Leferovich J., Gourevitch D., Sachadyn P., Zhang X.M., Clark L., Heber-Katz E. (2009). Retained features of embryonic metabolism in the adult MRL mouse. Mol. Genet. Metab..

[7] Ma S., Huang Q., Tominaga T., Liu C., Suzuki K. (2018). An 8-Week Ketogenic Diet Alternated Interleukin-6, Ketolytic and Lipolytic Gene Expression, and Enhanced Exercise Capacity in Mice. Nutrients.

[8] Ogborn D.I., McKay B.R., Crane J.D., Safdar A., Akhtar M., Parise G., Tarnopolsky M.A. (2015). Effects of age and unaccustomed resistance exercise on mitochondrial transcript and protein abundance in skeletal muscle of men. Am. J. Physiol. Regul. Integr. Comp. Physiol..

[9] Winckelmans E., Nawrot T.S., Tsamou M., Den Hond E., Baeyens W., Kleinjans J., Lefebvre W., Van Larebeke N., Peusens M., Plusquin M. (2017). Transcriptome-wide analyses indicate mitochondrial responses to particulate air pollution exposure. Environ. Health A Glob. Access Sci. Source.

[10] Naviaux R.K. (2014). Metabolic features of the cell danger response. Mitochondrion.

[11] Naviaux R.K. (2018). Metabolic features and regulation of the healing cycle-A new model for chronic disease pathogenesis and treatment. Mitochondrion.

[12] Quiros P.M., Prado M.A., Zamboni N., D’Amico D., Williams R.W., Finley D., Gygi S.P., Auwerx J. (2017). Multi-omics analysis identifies ATF4 as a key regulator of the mitochondrial stress response in mammals. J. Cell Biol..

[13] Nikkanen J., Forsstrom S., Euro L., Paetau I., Kohnz R.A., Wang L., Chilov D., Viinamaki J., Roivainen A., Marjamaki P. (2016). Mitochondrial DNA Replication Defects Disturb Cellular dNTP Pools and Remodel One-Carbon Metabolism. Cell Metab..

[14] Silva J.M., Wong A., Carelli V., Cortopassi G.A. (2009). Inhibition of mitochondrial function induces an integrated stress response in oligodendroglia. Neurobiol. Dis..

[15] Cameron J.L., Eagleson K.L., Fox N.A., Hensch T.K., Levitt P. (2017). Social Origins of Developmental Risk for Mental and Physical Illness. J. Neurosci. Off. J. Soc. Neurosci..

[16] Fredrickson B.L., Grewen K.M., Algoe S.B., Firestine A.M., Arevalo J.M., Ma J., Cole S.W. (2015). Psychological well-being and the human conserved transcriptional response to adversity. PLoS ONE.

[17] Picard M., McManus M.J., Gray J.D., Nasca C., Moffat C., Kopinski P.K., Seifert E.L., McEwen B.S., Wallace D.C. (2015). Mitochondrial functions modulate neuroendocrine, metabolic, inflammatory, and transcriptional responses to acute psychological stress. Proc. Natl. Acad. Sci. USA.

[18] Leday G.G.R., Vertes P.E., Richardson S., Greene J.R., Regan T., Khan S., Henderson R., Freeman T.C., Pariante C.M., Harrison N.A. (2018). Replicable and Coupled Changes in Innate and Adaptive Immune Gene Expression in Two Case-Control Studies of Blood Microarrays in Major Depressive Disorder. Biol. Psychiatry.

[19] Burnstock G., Knight G.E. (2017). Cell culture: Complications due to mechanical release of ATP and activation of purinoceptors. Cell Tissue Res..

[20] Burnstock G. (2018). The therapeutic potential of purinergic signalling. Biochem. Pharmacol..

[21] Burnstock G. (2016). Short- and long-term (trophic) purinergic signalling. Philos. Trans. R. Soc. Lond..

[22] De la Fuente I.M. (2015). Elements of the cellular metabolic structure. Front. Mol. Biosci..

[23] Jindrichova M., Kuzyk P., Li S., Stojilkovic S.S., Zemkova H. (2012). Conserved ectodomain cysteines are essential for rat P2X7 receptor trafficking. Purinergic Signal..

[24] Sussman I., Erecinska M., Wilson D.F. (1980). Regulation of cellular energy metabolism: The Crabtree effect. Biochim. Biophys. Acta.

[25] Dror E., Dalmas E., Meier D.T., Wueest S., Thevenet J., Thienel C., Timper K., Nordmann T.M., Traub S., Schulze F. (2017). Postprandial macrophage-derived IL-1beta stimulates insulin, and both synergistically promote glucose disposal and inflammation. Nat. Immunol..

[26] Heber-Katz E. (2017). Oxygen, Metabolism, and Regeneration: Lessons from Mice. Trends Mol. Med..

[27] Barros-Barbosa A.R., Oliveira A., Lobo M.G., Cordeiro J.M., Correia-de-Sa P. (2018). Under stressful conditions activation of the ionotropic P2X7 receptor differentially regulates GABA and glutamate release from nerve terminals of the rat cerebral cortex. Neurochem. Int..

[28] Xiong Y., Teng S., Zheng L., Sun S., Li J., Guo N., Li M., Wang L., Zhu F., Wang C. (2018). Stretch-induced Ca(2+) independent ATP release in hippocampal astrocytes. J. Physiol..

[29] Naviaux R.K. (2017). Antipurinergic therapy for autism-An in-depth review. Mitochondrion.

[30] Sakaki H., Tsukimoto M., Harada H., Moriyama Y., Kojima S. (2013). Autocrine regulation of macrophage activation via exocytosis of ATP and activation of P2Y11 receptor. PLoS ONE.

[31] Huang Z.L., Zhang Z., Qu W.M. (2014). Roles of adenosine and its receptors in sleep-wake regulation. Int. Rev. Neurobiol..

[32] He S., Sharpless N.E. (2017). Senescence in Health and Disease. Cell.

[33] Wang-Michelitsch J., Michelitsch T.M. (2015). Misrepair accumulation theory: A theory for understanding aging, cancer development, longevity, and adaptation. arXiv.

[34] Wang-Michelitsch J., Michelitsch T.M. (2015). Tissue fibrosis: A principal proof for the central role of Misrepair in aging. arXiv.

[35] Wang J., Michelitsch T., Wunderlin A., Mahadeva R. (2009). Aging as a consequence of misrepair--a novel theory of aging. arXiv.

[36] Zhao R., Liang D., Sun D. (2016). Blockade of Extracellular ATP Effect by Oxidized ATP Effectively Mitigated Induced Mouse Experimental Autoimmune Uveitis (EAU). PLoS ONE.

[37] Hultqvist M., Olsson L.M., Gelderman K.A., Holmdahl R. (2009). The protective role of ROS in autoimmune disease. Trends Immunol..

[38] Alvarez K., Vasquez G. (2017). Damage-associated molecular patterns and their role as initiators of inflammatory and auto-immune signals in systemic lupus erythematosus. Int. Rev. Immunol..

[39] Frenk S., Houseley J. (2018). Gene expression hallmarks of cellular ageing. Biogerontology.

[40] Chen W., Sandoval H., Kubiak J.Z., Li X.C., Ghobrial R.M., Kloc M. (2018). The phenotype of peritoneal mouse macrophages depends on the mitochondria and ATP/ADP homeostasis. Cell. Immunol..

[41] Liu P.S., Ho P.C. (2019). Determining Macrophage Polarization upon Metabolic Perturbation. Methods Mol. Biol..

[42] Van den Bossche J., O’Neill L.A., Menon D. (2017). Macrophage Immunometabolism: Where Are We (Going)?. Trends Immunol..

[43] Motori E., Puyal J., Toni N., Ghanem A., Angeloni C., Malaguti M., Cantelli-Forti G., Berninger B., Conzelmann K.K., Gotz M. (2013). Inflammation-induced alteration of astrocyte mitochondrial dynamics requires autophagy for mitochondrial network maintenance. Cell Metab..

[44] Roger A.J., Munoz-Gomez S.A., Kamikawa R. (2017). The Origin and Diversification of Mitochondria. Curr. Biol..

[45] Chen H., Chan D.C. (2017). Mitochondrial Dynamics in Regulating the Unique Phenotypes of Cancer and Stem Cells. Cell Metab..

[46] Giedt R.J., Fumene Feruglio P., Pathania D., Yang K.S., Kilcoyne A., Vinegoni C., Mitchison T.J., Weissleder R. (2016). Computational imaging reveals mitochondrial morphology as a biomarker of cancer phenotype and drug response. Sci. Rep..

[47] Singh B., Schoeb T.R., Bajpai P., Slominski A., Singh K.K. (2018). Reversing wrinkled skin and hair loss in mice by restoring mitochondrial function. Cell Death Dis..

[48] Atamna H., Tenore A., Lui F., Dhahbi J.M. (2018). Organ reserve, excess metabolic capacity, and aging. Biogerontology.

[49] Verkhratsky A., Burnstock G. (2014). Biology of purinergic signalling: Its ancient evolutionary roots, its omnipresence and its multiple functional significance. BioEssays News Rev. Mol. Cell. Dev. Biol..

[50] Burnstock G. (2015). Intracellular expression of purinoceptors. Purinergic Signal..

[51] Diaz-Hernandez J.I., Sebastian-Serrano A., Gomez-Villafuertes R., Diaz-Hernandez M., Miras-Portugal M.T. (2015). Age-related nuclear translocation of P2X6 subunit modifies splicing activity interacting with splicing factor 3A1. PLoS ONE.

[52] Dietz W.H. (1994). Critical periods in childhood for the development of obesity. Am. J. Clin. Nutr..

[53] Das J.K., Salam R.A., Thornburg K.L., Prentice A.M., Campisi S., Lassi Z.S., Koletzko B., Bhutta Z.A. (2017). Nutrition in adolescents: Physiology, metabolism, and nutritional needs. Ann. N. Y. Acad. Sci..

[54] Desbrow B., Burd N.A., Tarnopolsky M., Moore D.R., Elliott-Sale K.J. (2019). Nutrition for Special Populations: Young, Female, and Masters Athletes. Int. J. Sport Nutr. Exerc. Metab..

[55] Lopez-Lluch G., Irusta P.M., Navas P., de Cabo R. (2008). Mitochondrial biogenesis and healthy aging. Exp. Gerontol..

[56] Weir H.J., Yao P., Huynh F.K., Escoubas C.C., Goncalves R.L., Burkewitz K., Laboy R., Hirschey M.D., Mair W.B. (2017). Dietary Restriction and AMPK Increase Lifespan via Mitochondrial Network and Peroxisome Remodeling. Cell Metab..

[57] Rabol R., Svendsen P.F., Skovbro M., Boushel R., Haugaard S.B., Schjerling P., Schrauwen P., Hesselink M.K., Nilas L., Madsbad S. (2009). Reduced skeletal muscle mitochondrial respiration and improved glucose metabolism in nondiabetic obese women during a very low calorie dietary intervention leading to rapid weight loss. Metabolism.

[58] Muller M.J., Enderle J., Pourhassan M., Braun W., Eggeling B., Lagerpusch M., Gluer C.C., Kehayias J.J., Kiosz D., Bosy-Westphal A. (2015). Metabolic adaptation to caloric restriction and subsequent refeeding: The Minnesota Starvation Experiment revisited. Am. J. Clin. Nutr..

[59] Ewald C.Y., Castillo-Quan J.I., Blackwell T.K. (2018). Untangling Longevity, Dauer, and Healthspan in Caenorhabditis elegans Insulin/IGF-1-Signalling. Gerontology.

[60] Albert P.S., Riddle D.L. (1983). Developmental alterations in sensory neuroanatomy of the Caenorhabditis elegans dauer larva. J. Comp. Neurol..

[61] Lee D., Lee H., Kim N., Lim D.S., Lee J. (2017). Regulation of a hitchhiking behavior by neuronal insulin and TGF-beta signaling in the nematode Caenorhabditis elegans. Biochem. Biophys. Res. Commun..

[62] Lant B., Storey K.B. (2010). An overview of stress response and hypometabolic strategies in Caenorhabditis elegans: Conserved and contrasting signals with the mammalian system. Int. J. Biol. Sci..

[63] Androwski R.J., Flatt K.M., Schroeder N.E. (2017). Phenotypic plasticity and remodeling in the stress-induced Caenorhabditis elegans dauer. Wiley Interdiscip. Rev. Dev. Biol..

[64] D’Antona G., Ragni M., Cardile A., Tedesco L., Dossena M., Bruttini F., Caliaro F., Corsetti G., Bottinelli R., Carruba M.O. (2010). Branched-chain amino acid supplementation promotes survival and supports cardiac and skeletal muscle mitochondrial biogenesis in middle-aged mice. Cell Metab..

[65] Bonfili L., Cecarini V., Cuccioloni M., Angeletti M., Flati V., Corsetti G., Pasini E., Dioguardi F.S., Eleuteri A.M. (2017). Essential amino acid mixtures drive cancer cells to apoptosis through proteasome inhibition and autophagy activation. FEBS J..

[66] Corsetti G., Romano C., Pasini E., Marzetti E., Calvani R., Picca A., Flati V., Dioguardi F.S. (2017). Diet enrichment with a specific essential free amino acid mixture improves healing of undressed wounds in aged rats. Exp. Gerontol..

[67] Fielenbach N., Antebi A.C. (2008). elegans dauer formation and the molecular basis of plasticity. Genes Dev..

[68] Sender R., Fuchs S., Milo R. (2016). Revised Estimates for the Number of Human and Bacteria Cells in the Body. PLoS Biol..

[69] Renehan A.G., Booth C., Potten C.S. (2001). What is apoptosis, and why is it important?. BMJ.

[70] Spalding K.L., Arner E., Westermark P.O., Bernard S., Buchholz B.A., Bergmann O., Blomqvist L., Hoffstedt J., Naslund E., Britton T. (2008). Dynamics of fat cell turnover in humans. Nature.

[71] Spalding K.L., Bhardwaj R.D., Buchholz B.A., Druid H., Frisen J. (2005). Retrospective birth dating of cells in humans. Cell.

[72] Doherty T.J. (2003). Invited review: Aging and sarcopenia. J. Appl. Physiol..

[73] Prescott L.F. (1966). The normal urinary excretion rates of renal tubular cells, leucocytes and red blood cells. Clin. Sci..

[74] Rinkevich Y., Montoro D.T., Contreras-Trujillo H., Harari-Steinberg O., Newman A.M., Tsai J.M., Lim X., Van-Amerongen R., Bowman A., Januszyk M. (2014). In vivo clonal analysis reveals lineage-restricted progenitor characteristics in mammalian kidney development, maintenance, and regeneration. Cell Rep..

[75] Kim I.H., Kisseleva T., Brenner D.A. (2015). Aging and liver disease. Curr. Opin. Gastroenterol..

[76] Bergmann O., Bhardwaj R.D., Bernard S., Zdunek S., Barnabe-Heider F., Walsh S., Zupicich J., Alkass K., Buchholz B.A., Druid H. (2009). Evidence for cardiomyocyte renewal in humans. Science.

[77] Perl S., Kushner J.A., Buchholz B.A., Meeker A.K., Stein G.M., Hsieh M., Kirby M., Pechhold S., Liu E.H., Harlan D.M. (2010). Significant human beta-cell turnover is limited to the first three decades of life as determined by in vivo thymidine analog incorporation and radiocarbon dating. J. Clin. Endocrinol. Metab..

[78] Fox J.P., Hall C.E., Cooney M.K., Luce R.E., Kronmal R.A. (1972). The Seattle virus watch. II. Objectives, study population and its observation, data processing and summary of illnesses. Am. J. Epidemiol..

[79] Milholland B., Dong X., Zhang L., Hao X., Suh Y., Vijg J. (2017). Differences between germline and somatic mutation rates in humans and mice. Nat. Commun..

[80] Peterson S.E., Westra J.W., Paczkowski C.M., Chun J. (2008). Chromosomal mosaicism in neural stem cells. Methods Mol. Biol..

[81] Muotri A.R., Chu V.T., Marchetto M.C., Deng W., Moran J.V., Gage F.H. (2005). Somatic mosaicism in neuronal precursor cells mediated by L1 retrotransposition. Nature.

[82] Romer C., Singh M., Hurst L.D., Izsvak Z. (2017). How to tame an endogenous retrovirus: HERVH and the evolution of human pluripotency. Curr. Opin. Virol..

[83] Harris S.A., Harris E.A. (2015). Herpes Simplex Virus Type 1 and Other Pathogens are Key Causative Factors in Sporadic Alzheimer’s Disease. J. Alzheimer’s Dis. JAD.

[84] Bornhofft K.F., Goldammer T., Rebl A., Galuska S.P. (2018). Siglecs: A journey through the evolution of sialic acid-binding immunoglobulin-type lectins. Dev. Comp. Immunol..

[85] Schwarz F., Pearce O.M., Wang X., Samraj A.N., Laubli H., Garcia J.O., Lin H., Fu X., Garcia-Bingman A., Secrest P. (2015). Siglec receptors impact mammalian lifespan by modulating oxidative stress. Elife.

[86] Stanczak M.A., Siddiqui S.S., Trefny M.P., Thommen D.S., Boligan K.F., von Gunten S., Tzankov A., Tietze L., Lardinois D., Heinzelmann-Schwarz V. (2018). Self-associated molecular patterns mediate cancer immune evasion by engaging Siglecs on T cells. J. Clin. Investig..

[87] Bower N.I., Hogan B.M. (2018). Brain drains: New insights into brain clearance pathways from lymphatic biology. J. Mol. Med..

[88] Schubert D., Currais A., Goldberg J., Finley K., Petrascheck M., Maher P. (2018). Geroneuroprotectors: Effective Geroprotectors for the Brain. Trends Pharmacol. Sci..

[89] Mizutani T., Ishizaka A., Furuichi Y. (2015). The Werner Protein Acts as a Coactivator of Nuclear Factor kappaB (NF-kappaB) on HIV-1 and Interleukin-8 (IL-8) Promoters. J. Biol. Chem..

[90] Huang Y., Li W., Su Z.Y., Kong A.N. (2015). The complexity of the Nrf2 pathway: Beyond the antioxidant response. J. Nutr. Biochem..

[91] Li B., Iglesias-Pedraz J.M., Chen L.Y., Yin F., Cadenas E., Reddy S., Comai L. (2014). Downregulation of the Werner syndrome protein induces a metabolic shift that compromises redox homeostasis and limits proliferation of cancer cells. Aging Cell.

[92] Blanc E.M., Bruce-Keller A.J., Mattson M.P. (1998). Astrocytic gap junctional communication decreases neuronal vulnerability to oxidative stress-induced disruption of Ca2+ homeostasis and cell death. J. Neurochem..

[93] Duchen M.R. (2000). Mitochondria and calcium: From cell signalling to cell death. J. Physiol..

[94] Camacho-Pereira J., Tarrago M.G., Chini C.C.S., Nin V., Escande C., Warner G.M., Puranik A.S., Schoon R.A., Reid J.M., Galina A. (2016). CD38 Dictates Age-Related NAD Decline and Mitochondrial Dysfunction through an SIRT3-Dependent Mechanism. Cell Metab..

[95] Igwe O.J., Filla M.B. (1997). Aging-related regulation of myo-inositol 1,4,5-trisphosphate signal transduction pathway in the rat striatum. Brain Res. Mol. Brain Res..

[96] Gomes A.P., Price N.L., Ling A.J., Moslehi J.J., Montgomery M.K., Rajman L., White J.P., Teodoro J.S., Wrann C.D., Hubbard B.P. (2013). Declining NAD(+) induces a pseudohypoxic state disrupting nuclear-mitochondrial communication during aging. Cell.

[97] Chini C.C.S., Tarrago M.G., Chini E.N. (2017). NAD and the aging process: Role in life, death and everything in between. Mol. Cell. Endocrinol..

[98] Zhang H., Ryu D., Wu Y., Gariani K., Wang X., Luan P., D’Amico D., Ropelle E.R., Lutolf M.P., Aebersold R. (2016). NAD(+) repletion improves mitochondrial and stem cell function and enhances life span in mice. Science.

[99] Xu M., Pirtskhalava T., Farr J.N., Weigand B.M., Palmer A.K., Weivoda M.M., Inman C.L., Ogrodnik M.B., Hachfeld C.M., Fraser D.G. (2018). Senolytics improve physical function and increase lifespan in old age. Nat. Med..

[100] Go Y.M., Fernandes J., Hu X., Uppal K., Jones D.P. (2018). Mitochondrial network responses in oxidative physiology and disease. Free Radic. Biol. Med..

[101] Salminen A., Kaarniranta K., Kauppinen A. (2017). Integrated stress response stimulates FGF21 expression: Systemic enhancer of longevity. Cell Signal..

[102] Pakos-Zebrucka K., Koryga I., Mnich K., Ljujic M., Samali A., Gorman A.M. (2016). The integrated stress response. EMBO Rep..

[103] Naviaux R.K. (2012). Oxidative shielding or oxidative stress?. J. Pharmacol. Exp. Ther..

[104] Wang-Michelitsch J., Michelitsch T. (2015). Aging as a process of accumulation of Misrepairs. arXiv.

[105] Hernandez-Segura A., de Jong T.V., Melov S., Guryev V., Campisi J., Demaria M. (2017). Unmasking Transcriptional Heterogeneity in Senescent Cells. Curr. Biol..

[106] Kumari J., Hussain M., De S., Chandra S., Modi P., Tikoo S., Singh A., Sagar C., Sepuri N.B., Sengupta S. (2016). Mitochondrial functions of RECQL4 are required for the prevention of aerobic glycolysis-dependent cell invasion. J. Cell Sci..

[107] Gupta S., De S., Srivastava V., Hussain M., Kumari J., Muniyappa K., Sengupta S. (2014). RECQL4 and p53 potentiate the activity of polymerase gamma and maintain the integrity of the human mitochondrial genome. Carcinogenesis.

[108] Croteau D.L., Singh D.K., Hoh Ferrarelli L., Lu H., Bohr V.A. (2012). RECQL4 in genomic instability and aging. Trends Genet..

[109] Hupin D., Roche F., Gremeaux V., Chatard J.C., Oriol M., Gaspoz J.M., Barthelemy J.C., Edouard P. (2015). Even a low-dose of moderate-to-vigorous physical activity reduces mortality by 22% in adults aged >/=60 years: A systematic review and meta-analysis. Br. J. Sports Med..

[110] Gries K.J., Raue U., Perkins R.K., Lavin K.M., Overstreet B.S., D’Acquisto L.J., Graham B., Finch W.H., Kaminsky L.A., Trappe T.A. (2018). Cardiovascular and Skeletal Muscle Health with Lifelong Exercise. J. Appl. Physiol..

[111] Radak Z., Torma F., Berkes I., Goto S., Mimura T., Posa A., Balogh L., Boldogh I., Suzuki K., Higuchi M. (2019). Exercise effects on physiological function during aging. Free Radic. Biol. Med..

[112] Fan W., Evans R.M. (2017). Exercise Mimetics: Impact on Health and Performance. Cell Metab..

[113] Yu M., Tsai S.F., Kuo Y.M. (2017). The Therapeutic Potential of Anti-Inflammatory Exerkines in the Treatment of Atherosclerosis. Int. J. Mol. Sci..

[114] Whitham M., Parker B.L., Friedrichsen M., Hingst J.R., Hjorth M., Hughes W.E., Egan C.L., Cron L., Watt K.I., Kuchel R.P. (2018). Extracellular Vesicles Provide a Means for Tissue Crosstalk during Exercise. Cell Metab..

[115] Safdar A., Tarnopolsky M.A. (2018). Exosomes as Mediators of the Systemic Adaptations to Endurance Exercise. Cold Spring Harb. Perspect. Med..

[116] Jesko H., Stepien A., Lukiw W.J., Strosznajder R.P. (2018). The Cross-Talk Between Sphingolipids and Insulin-Like Growth Factor Signaling: Significance for Aging and Neurodegeneration. Mol. Neurobiol..

[117] Trayssac M., Hannun Y.A., Obeid L.M. (2018). Role of sphingolipids in senescence: Implication in aging and age-related diseases. J. Clin. Investig..

[118] Jazwinski S.M. (2015). Mitochondria to nucleus signaling and the role of ceramide in its integration into the suite of cell quality control processes during aging. Ageing Res. Rev..

[119] Fakouri N.B., Hansen T.L., Desler C., Anugula S., Rasmussen L.J. (2019). From powerhouse to perpetrator—mitochondria in health and disease. Biology.

